# Global Transmission, Prevention, Control, and Treatment of Mpox Virus in 2025: A Comprehensive Review from Infection Mechanisms to Vaccine Development

**DOI:** 10.3390/vaccines13101071

**Published:** 2025-10-20

**Authors:** Quan Quan, Nan Wu, Ying-Hua Luo, Yan-Jun Tang, Yan-Zhi Liu, Xi-Chun Huang, Jun-Hao Li, Wan-Xia Ren, Cheng-Hao Jin

**Affiliations:** 1 Department of Biochemistry and Molecular Biology, College of Life Science and Biotechnology, Heilongjiang Bayi Agricultural University, Daqing 163319, China; quanquan@byau.edu.cn (Q.Q.); wunan0925@byau.edu.cn (N.W.); liuyanzhi@byau.edu.cn (Y.-Z.L.); huangxichun@byau.edu.cn (X.-C.H.); lijunhao1018@byau.edu.cn (J.-H.L.); 2 Department of Food Science and Engineering, College of Food Science, Heilongjiang Bayi Agricultural University, Daqing 163319, China; tttyyjj@126.com; 3 National Coarse Cereals Engineering Research Center, Daqing 163319, China; 4 Department of Grass Science, College of Animal Science and Veterinary Medicine, Heilongjiang Bayi Agricultural University, Daqing 163319, China; luoyinghua202208@163.com; 5 Hospital Clinical Laboratory, Heilongjiang Bayi Agricultural University, Daqing 163319, China

**Keywords:** Mpox, infection mechanism, pathogenic mechanism, immune escape, vaccine research and development

## Abstract

The World Health Organization (WHO) declared the mpox (MPX) outbreak a public health emergency of international concern (PHEIC) on 23 July 2022, and 14 August 2024, respectively, underscoring the confirmed and concerning global spread of the disease. A gap exists in our fundamental understanding of the mpox virus (MPXV), despite its genetic relatedness to the variola virus (VARV). This knowledge deficit is evident in the performance of current medical countermeasures; vaccines and antiviral therapies adapted from smallpox programs demonstrate only partial efficacy and are constrained by issues of safety and suboptimal effectiveness against MPXV. In this context, the development of MPX-specific vaccines and antiviral drugs has become a critical priority in the global effort to combat MPX. However, MPXV employs multiple strategies to evade host immune responses, such as producing specific and poxvirus homologous proteins that suppress both innate immunity (including the six principal innate immune signaling pathways and antiviral strategies, notably the interferon [IFN] pathway) and adaptive immunity, thereby complicating vaccine and drug development. Insights from research on vaccinia virus (VACV) and VARV may inform the investigation of MPXV pathogenesis and immune evasion mechanisms. Drawing on relevant scientific literature, this review systematically examines key aspects of MPX infection, pathogenicity, and immune evasion, as well as the coordination between innate and adaptive immune responses. Furthermore, this review elucidates the current application and deployment landscape of the three principal therapeutics and three major vaccines for MPX, aiming to provide a theoretical foundation for future research and development of vaccines and targeted antiviral agents.

## 1. Introduction

**Figure 1:**
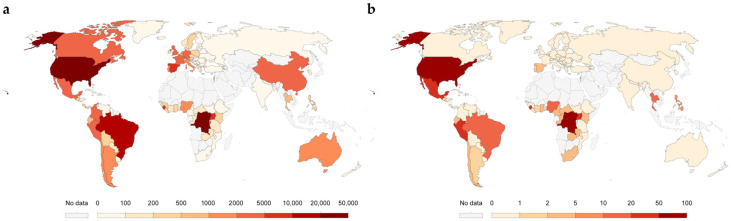
Global cumulative case report distribution of MPX. (**a**) Global cumulative case distribution. (**b**) Global distribution of death cases. Note: The data is from the WHO (Global Mpox Trends) and is updated as of 31 July 2025. Output the chart through Our World in Data (Supplementary Materials, https://ourworldindata.org/mpox#explore-our-data-on-mpox, accessed on 31 July 2025) website. The cases shown in this picture are only laboratory confirmed cases. 2985 cases for the Democratic Republic of the Congo were reported without a date and are not included in this chart. The WHO defines an MPX death as any death of a person with probable or confirmed MPX unless the death is attributed to trauma.

MPX is a zoonotic disease caused by infection with the MPXV, a member of the orthopoxvirus genus. As of 31 July 2025, a total of 34,386 confirmed cases and 138 fatalities have been reported across 84 countries worldwide (Figure 1). The global outbreak from 2022 to 2023 was primarily driven by clade IIb strains of the West African branch [1]. In contrast, the 2024 MPX outbreak was caused by clade I strains of the Central African branch, which exhibit a significantly higher case fatality rate (CFR) of up to 10%, compared to 1–3% for clade IIa and 0.2–1% for clade IIb [2]. In response, the WHO designated the MPX outbreak as a PHEIC on 23 July 2022, and again on 14 August 2024 [3,4].

Due to clade-specific genetic variations, key protein deletions/truncations, and structural differences in the genome between MPXV and other orthopoxviruses, no specific antiviral drugs or vaccines against MPX have been developed to date. Currently available vaccines are associated with limitations such as adverse effects, inequitable global distribution, and insufficient mechanistic understanding [5]. With regard to therapeutic agents, genetic variability and widespread transmission have facilitated viral mutations, leading to alterations in crucial MPXV proteins (e.g., E5R, K1R, A47L). These mutations may undermine the efficacy of compounds designed to target antigenic epitopes shared with other orthopoxviruses [6,7,8]. Consequently, the development of effective treatments and vaccines against MPX continues to face considerable challenges.

## 2. The History of MPX

The natural reservoir host of MPXV remains undetermined; however, the virus primarily infects wild rodents in tropical rainforests within its endemic regions. The name “mpox” originated from the initial detection of the disease in 1958 in cynomolgus monkeys housed in a laboratory in Copenhagen, Denmark [9]. Prior to 1970, MPX was believed to infect only nonhuman primates such as monkeys and apes. However, in August of that year, the first human case of MPX was reported in a 9-month-old boy in the Democratic Republic of the Congo, confirming the zoonotic and pathogenic potential of MPXV in humans [10]. Subsequently, the majority of documented human cases caused by the Central African clade I have been reported in the Congo Basin region. In 2003, an outbreak in the United States, attributed to the West African clade IIa strain and linked to imported infected rodents, marked the first occurrence of MPX outside endemic African regions [11].

## 3. The Branches of and Differences in MPXV

MPXV is a large, double stranded DNA virus belonging to the genus orthopoxvirus within the family Poxviridae. Its closest known relatives include the VARV and the cowpox virus (CPXV). Since its initial identification, MPXV has been classified into two distinct clades: Clade I, associated with the Central African (Congo Basin) region, and Clade II, originating from West Africa [12,13]. The MPXV genome is notably long and complex, ranging from approximately 196 kbp to 211 kbp. Clade I genomes span 196–199 kbp, while Clade II genomes range from 196 kbp to 211 kbp. The genome harbors approximately 223 nonoverlapping open reading frames (ORFs), encoding 181 proteins. It consists of a conserved central region flanked by inverted terminal repeats (ITRs) that vary in length from 6.5 kbp to 17.5 kbp. The linear DNA molecule features covalently closed hairpin termini, lacking free 3′ or 5′ ends. Notable genetic differences between clades include the absence of a vaccinia virus Copenhagen (VACV-Cop) E5R homolog and truncations in the homologs of K1R, VACV-Cop A47L, and VACV-Cop B11R in Clade I. In Clade II, four genes: D14L, D15L, D16L, and D17L are absent, and three genes: D4L, B14L, and B15L are truncated [14,15].

## 4. The Transmission of MPX

**Figure 2:**
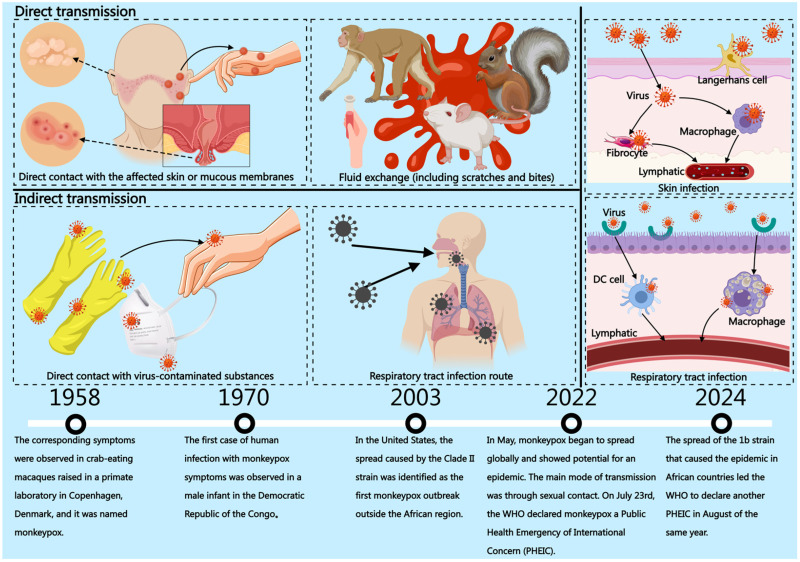
The discovery of MPX and its landmark historical events. Close contact is a major way of direct transmission. This process involves direct contact with the patient’s infected area, as well as sexual behaviors (such as male-to-male sexual activities) and body fluid exchange caused by scratches or bites from animals carrying the virus. Indirect transmission is not direct contact with the patient, but contact of the skin or mucous membranes with the virus and its contaminants. The pictures respectively show the process of virus infection through the skin and mucous membrane pathways. This picture was drawn using the Medpeer (https://www.medpeer.cn/product/index/product, accessed on 2 September 2025) website.

MPX transmission occurs primarily through direct contact (intimate interactions) and indirect routes (exposure to contaminated fomites) (Figure 2). Human-to-human transmission often results from close contact with respiratory secretions, skin or genital lesions, prolonged face-to-face exposure, or contaminated materials such as bedding and clothing. Zoonotic transmission involves direct contact with infected animals through lesions or bodily fluids, scratches or bites, consumption of infected animal meat, or exposure to contaminated objects. Indirect transmission mainly occurs via contact with respiratory mucosal surfaces [8,16]. Although MPXV is a large virus unable to cross the placental pore, vertical transmission of MPX has been documented. Infection during pregnancy may lead to abortion or stillbirth, and viral DNA has been detected in the semen of infected males [17]. In endemic African regions, children represent a major affected demographic. In contrast, in nonendemic areas such as Europe and the Americas, case distribution shows a pronounced gender disparity, with men who have sex with men (MSM) constituting a key transmission group [18]. Given that MPX spreads predominantly through close physical contact, individuals with underlying health conditions or immunocompromised states are at higher risk of infection and severe disease. Thus, high exposure groups, such as healthcare workers, sex workers, and those in frequent contact with infected individuals or wildlife, as well as vulnerable populations including newborns, children, the elderly, and pregnant women, are particularly susceptible to MPX [19,20]. In summary, increasing global mobility amplifies the transmission potential of MPX through contact, respiratory droplets, and contaminated objects.

## 5. Mutation and Evolution of MPXV

Despite possessing a double-stranded DNA genome with proofreading 3′-5′ exonuclease activity, which theoretically confers a low mutation rate, MPXV has exhibited an unexpectedly high evolutionary rate in recent outbreaks. The global substitution rate of MPXV in 2022 was estimated at 38.63 mutations per genome per year [21]. Comparative genomic analyses revealed an average of 50 single-nucleotide polymorphisms (SNPs) between 2022 outbreak strains and related viruses from 2018 to 2019. Among these, 26 GA > AA and 15 TC > TT substitutions indicated a strong mutational bias, far exceeds the evolutionary rates predicted by the three molecular clock models: strict molecular clock (Strict), uncorrelated log-normal molecular clock (UCLN), and uncorrelated exponential molecular clock (UCED). Given the 96.3% nucleotide sequence identity between MPXV and VARV, it is hypothesized that additional factors accelerate MPXV mutagenesis [22]. Growing evidence suggests that interactions between MPX inhibitor of complement enzymes (MOPICE) and host innate immune factors, particularly apolipoprotein B mRNA editing catalytic polypeptide-like 3 (APOBEC3), serve as a major driver of MPXV evolution. Sampling of clade IIb MPXV sequences between 2017 and 2022 revealed a significant enrichment of APOBEC3-mediated G-to-A mutations throughout lineage A. Another study indicated that APOBEC3 preferentially targets cruciform DNA secondary structures formed by inverted repeat (IR) regions. Notably, 63.9% of the more than 50 SNPs identified in the 2022 outbreak strains were located within IR regions of the 2018 reference genome. These findings align with the observed nucleotide mutation bias in MPXV and provide mechanistic insight into its accelerated evolution [23,24].

## 6. The Infection Mechanism of MPX

**Figure 3:**
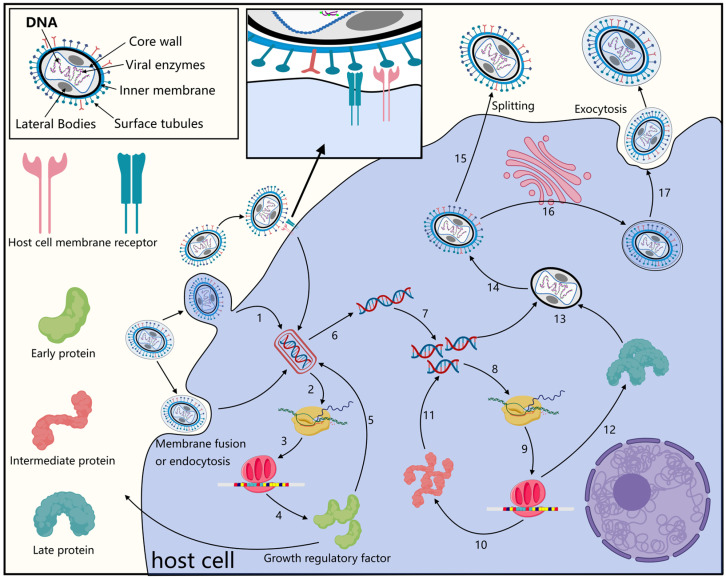
The life cycle diagram of MPXV. MPXV enters the cell through endocytosis and fuses with the membrane to carry out primary disintegration and initiate the transcriptional expression of early genes to synthesize early proteins. The early proteins induce the expression of viral genome genes and participate in the regulation of the immune and growth signals of the host cell, blocking or activating corresponding signaling pathways, inhibiting the immune response and the process of programmed cell death. This is the key to MPXV’s immune evasion. The late proteins are the main components of the virus’s composition structure. After the virus assembly is completed, depending on whether it passes through the Golgi apparatus, it can be classified into two forms: mature virion (MV) and enveloped virion (EV). Then, the two virus particles escape from the cell through lysis or exocytosis. This picture was drawn using the Medpeer (https://www.medpeer.cn/product/index/product, accessed on 4 September 2025) website. (1: Viral Uncoating; 2, 8: Transcription; 3, 9: Translation; 4: Early Protein Synthesis; 10: Mid-phase protein synthesis; 5, 11: Catalysis; 6: Genome Release; 7: DNA Replication; 12: Late Protein Synthesis; 13, 14: Virus Particle Assembly; 15, 17: Virus Particle Release; 16: Envelopment).

MPXV shares a conserved mechanism of viral adsorption and entry with other orthopoxviruses, such as CPXV [25]. Following attachment to the cell surface via specific interactions, MPXV enters the host cytoplasm through membrane fusion, macropinocytosis, or endocytosis. This process depends on an entry fusion complex composed of at least 11 conserved poxviral proteins (e.g., A28 and A21) and several host auxiliary factors [16]. Notably, the conserved oligomeric golgi (COG) complex, a vesicular tethering complex comprising eight host proteins (COG1-COG8) plays a critical role in viral entry and fusion, with COG4 and COG7 implicated as central mediators [26]. Essential viral envelope proteins required for poxvirus entry include L1, F9, A28, A27, L5, and H2 [27]. MPXV also expresses additional proteins, such as A29L, A35R, B6R, M1R, E8L, and H3L, which contribute to virus–host membrane fusion. In the absence of specific virus receptor engagement, MPXV entry may involve interactions with host cell surface glycosaminoglycans (e.g., heparan sulfate and chondroitin sulfate), participation in intracellular enveloped virion (IEV) envelope association (Figure 3), and interactions with viral proteins such as A33 [28]. Interestingly, androgen receptor (AR) signaling pathway genes exhibit overlap with genes implicated in HIV infection and inflammatory responses, suggesting AR signaling may represent a potential therapeutic target against MPXV infection [29]. In a separate study using MPXV infected HeLa cells, key differentially expressed hub genes included IER3, IFIT2, IL11, ZC3H12A, EREG, IER2, NFKBIE, FST, IFIT1, and AREG. Among these, IFIT1 and IFIT2 were significantly downregulated [30].

## 7. The Pathogenic Mechanism of MPX

Currently, the cellular and tissue tropism of MPXV remains poorly defined. However, emerging evidence suggests that the MPX complement enzyme inhibitor (MOPICE) and complement control proteins (CCPs) are implicated in modulating viral tropism [31]. MPXV primarily infects oral and respiratory mucosal epithelia, where it initiates papule formation, followed by dissemination to regional lymph nodes and potential spread to secondary organs such as the spleen and liver [32]. During early infection, MPXV replicates locally within dendritic cells and macrophages, facilitating transit via the lymphatic system to draining lymph nodes. This process is detected by host innate immune receptors, including Toll-like receptors (TLRs) which activate downstream signaling pathways such as NF-κB, leading to elevated production of proinflammatory cytokines including TNF-α, IL-1β, and IL-6. These mediators contribute to systemic symptoms including fever, myalgia, and hyperalgesia [33]. Within lymph nodes, viral replication stimulates antigen-presenting cells (APCs) to release chemokines such as monocyte chemoattractant protein 1 (MCP-1), recruiting monocytes and enhancing immune cell infiltration. This amplifies inflammatory cytokine release and promotes vasodilation and tissue edema. Meanwhile, viral replication in keratinocytes results in lytic cell death and vesicle formation. Collectively, these mechanisms underpin the hallmark clinical manifestations of MPXV infection, namely lymphadenopathy and cutaneous rash [34,35,36].

## 8. MPX and Immunity

The host defense against viral infection involves both innate and adaptive immune responses. During the initial phase of infection, innate immune cells recognize pathogen-associated molecular patterns via pattern recognition receptors (PRRs), leading to the activation of downstream signaling pathways and upregulated transcription of inflammatory cytokines and chemokines [37]. These cytokines not only initiate the transcription of antiviral genes within infected and neighboring cells but also recruit additional immune cells, thereby establishing an antiviral state. Concurrently, T cells become activated and proliferate into effector cells through direct cytokine stimulation and indirect recognition of antigenic peptides presented by major histocompatibility complex (MHC) molecules on antigen presenting cells. This process effectively bridges innate and adaptive immunity [38,39].

**Figure 4:**
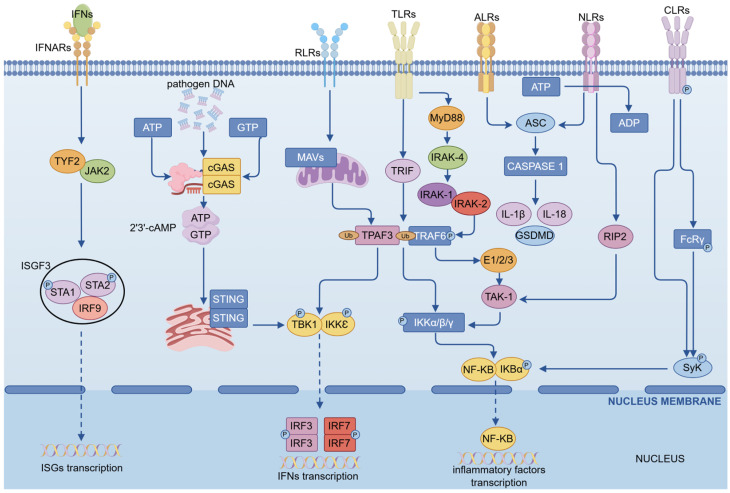
The six major innate immune pathway signaling pathways in the human body. The six innate immune pathways serve as the first line of defense when cells are infected by viruses, activating corresponding signaling pathways in response to different types or structures of pathogens. Although current reports do not indicate that all PRRs are involved in the recognition of MPXV, almost all innate immune pathways are involved in the inflammatory response and the IFN pathway. NF-κB is a key intermediate factor in the innate immune pathways. The figure shows some key targets and network connections of the six innate immune pathways and the IFN pathway. This picture was drawn using the Figdraw (https://www.figdraw.com/static/index.html#/, accessed on 12 September 2025) website. (p: Phosphorylation; Ub: Ubiquitination).

PRRs are essential components in the activation of the innate immune response. They primarily encompass TLRs, C-type lectin receptors (CLRs), RIG-I-like receptors (RLRs), NOD-like receptors (NLRs), AIM2-like receptors (ALRs), and intracellular DNA sensors such as cyclic GMP-AMP synthase (cGAS) [37,40]. These receptors specifically recognize distinct pathogen associated molecular patterns, including nucleic acids, carbohydrates, and structural proteins, thereby initiating downstream signaling cascades. The innate immune signaling network involves a series of core transcription factors, such as NF-κB and interferon regulatory factors (IRFs), which drive inflammatory responses and upregulate various cytokines and chemokines (Figure 4). These mediators recruit and activate additional immune cells, thereby bridging innate and adaptive immunity and establishing an overall antiviral state in the host [41,42]. Clearance of viruses and infected cells is primarily achieved through two mechanisms: (1) effector immune cells, such as CD8^+^ T cells, mediate targeted lysis or apoptosis of infected cells, and (2) infected cells themselves undergo programmed cell death, including apoptosis and pyroptosis upon activation of innate immune pathways. However, MPXV encodes numerous immunomodulatory proteins that suppress the activation of key immune signaling pathways. For instance, viral IL-1β binding proteins (vIL-1βBPs) and vIL-18BPs neutralize the activity of IL-1β and IL-18, thereby attenuating pyroptosis and inflammatory responses [43]. Additionally, orthopoxvirus major histocompatibility complex class I-like protein (OMCP) competitively binds to the NKG2D receptor, inhibiting natural killer (NK) cell activation and downregulating MHC class I expression to evade T cell recognition [16]. In summary, the potent suppression of both innate and adaptive immune responses by MPXV encoded proteins represents a major obstacle to the development of effective vaccines and therapeutics.

## 9. Clinical Diagnostic Methods and Principles of MPX

Currently, following WHO recommendations, nucleic acid amplification tests (NAATs) have been incorporated for molecular detection of the virus. These tests fundamentally amplify target nucleic acids from pathogens to achieve high throughput detection. Primary NAAT methodologies include reverse transcription polymerase chain reaction (RT-PCR), nicking endonuclease amplification reaction (NEAR), transcription-mediated amplification (TMA), loop-mediated isothermal amplification (LAMP), helicase-dependent amplification (HDA), clustered regularly interspaced short palindromic repeat (CRISPR)-based assays, and strand displacement amplification (SDA). Among these, real-time quantitative polymerase chain reaction (qPCR) is regarded as the preferred method for MPX detection due to its high throughput, sensitivity, and rapid turnaround time [16]. Established MPXV molecular targets include the envelope protein gene (B6R), B7R gene, DNA polymerase gene (E9L), complement binding protein gene (C3L), DNA directed RNA polymerase subunit 18 (RPO18) gene, F3L, and N3R [11,44]. Recombinase polymerase amplification (RPA), with a limit of detection (LOD) of 16 DNA molecules per microliter, has been demonstrated to be a more sensitive alternative to real time qPCR [45]. Beyond NAATs, additional methods such as viral isolation by cell culture and serological assays for antibody detection are also employed. Commonly used techniques include enzyme linked immunosorbent assay (ELISA) and indirect immunofluorescence assay (IFA). However, these approaches are generally limited by longer turnaround times, low sensitivity during the early window of infection, potential cross-reactivity with other orthopoxviruses leading to false positives, and overall lower specificity [46,47].

## 10. Types and Principles of Antiviral Drugs

To date, the primary therapeutics employed for the treatment of MPX include Tecovirimat, Cidofovir, and Brincidofovir [1,16,36]. Among these, Tecovirimat functions by targeting the viral VP37 protein, thereby inhibiting the formation of the viral envelope and preventing viral egress from host cells. It does not, however, interfere with intracellular viral replication. Due to its favorable safety profile, it is recommended as a first line treatment for pregnant and lactating patients [48].

Cidofovir acts through inhibition of viral DNA polymerase activity, suppressing viral replication and transcription. It received approval from the U.S. Food and Drug Administration (FDA) in 1996 for the treatment of cytomegalovirus retinitis in immunocompromised individuals, such as AIDS patients, and has demonstrated efficacy against various DNA viruses, including orthopoxviruses [49,50].

Brincidofovir, a lipid conjugated prodrug of Cidofovir, offers improved oral bioavailability and enhanced cellular uptake. Upon oral administration, it is converted into Cidofovir and subsequently phosphorylated to its active metabolite, cidofovir diphosphate (CDV-PP), which inhibits viral DNA polymerase or incorporates into the viral DNA strand as an acyclic nucleotide analog to terminate DNA synthesis [51]. Approved by the FDA in 2021 for smallpox treatment, it exhibits lower nephrotoxicity compared to Cidofovir [8].

Notwithstanding their clinical utility, these agents present several limitations: Tecovirimat shows variable and suboptimal effectiveness in MPX patients with significant interindividual variability; Cidofovir is associated with dose-dependent renal toxicity; and Brincidofovir may cause notable adverse effects, while its efficacy against MPX in humans requires further validation.

## 11. Types and Application Status of Vaccines

Vaccines against MPX are classified by the WHO into three categories: replicating (e.g., ACAM2000), minimally replicating (e.g., LC16m8), and nonreplicating (e.g., MVA-BN, currently in use) [1,52,53]. Smallpox vaccines confer approximately 85% cross protective immunity against MPX. However, first- and second-generation smallpox vaccines (e.g., those based on replicating VACV) have been associated with a range of adverse events in vaccinees [54]. MVA-BN, a third-generation attenuated nonreplicating vaccine approved by the FDA in 2019, is recommended for human prophylactic use due to its more favorable safety profile compared to ACAM2000 [55]. LC16m8, another attenuated vaccine candidate developed through serial passage at low temperature, exhibits reduced neurovirulence and is currently licensed only in Japan [56].

The Dryvax vaccine was one of the most long-standing vaccines against VARV. It was produced from calf lymph and contained live, infectious VACV along with trace amounts of several antibiotics, such as streptomycin sulfate, chlortetracycline hydrochloride, and neomycin sulfate. Successful vaccination was indicated by the formation of a vesicle or pustule at the intradermal inoculation site on the upper arm [57]. Dryvax confers approximately 85% long term cross protection against MPX. As it consisted of a heterogeneous mixture of VACV clones, it offered broad immunogenic coverage; however, this composition also increased the risks of co-infection, mutational events, and vaccine associated adverse events [58]. Due to these safety concerns, Dryvax was eventually superseded by newer vaccines such as ACAM2000 and MVA-BN. Notably, Dryvax elicits a stronger antibody response compared to the clonally derived ACAM2000. The two vaccines can be serologically distinguished based on their differential antibody reactivity to 11 viral proteins, including A13L, G5R, J6R, B19R, A38L, A26L, I1L, I3L, D8L, C3L, and A10L [59].

ACAM2000 is derived from a single clonal viral isolate of the first-generation smallpox vaccine Dryvax. It is a live virus vaccine based on VACV and was originally developed for protection against smallpox (variola). On 29 August 2024, it was approved by the FDA for immunization against MPX [60]. Leveraging the tropism of orthopoxviruses for immune cells, such as Langerhans cells via lymphatic drainage, ACAM2000 inoculation typically results in the formation of a localized vesicle or pustule (commonly referred to as a “take”) at the vaccination site, which serves as a marker of successful vaccine uptake. As a replication-competent vaccine, the virus can replicate within host cells, and antigens are presented by APCs, thereby activating adaptive immunity and establishing long term immunological memory [61,62]. Animal studies have demonstrated high levels of protection against MPXV challenge following ACAM2000 vaccination. However, in humans, its use is associated with potential adverse effects including cardiac complications, severe pain at the injection site, and significant cutaneous reactions. It is also contraindicated during pregnancy due to risks of severe side effects to both mother and fetus. Owing to these safety concerns, ACAM2000 is currently recommended only for individuals at high risk of exposure to orthopoxviruses [63].

MVA-BN is a nonreplicating live viral vaccine based on the modified vaccinia ankara (MVA) platform, developed for protection against both smallpox and MPX. As a third-generation attenuated VACV vaccine, it received approval from the FDA in 2019 [64]. Due to its inability to replicate in human cells, MVA-BN does not cause vaccine-associated viral infection symptoms, making it suitable for immunocompromised adults and other high-risk populations. It is also considered safe for administration during pregnancy. A two-dose regimen administered subcutaneously or intradermally at an interval of four weeks is required to establish robust vaccine-induced immunity [65]. Studies have reported that MVA-BN confers protection against MPX infection, with immunogenicity comparable to that of Dryvax at both 14 and 180 days postvaccination [66,67]. Current evidence suggests a vaccine effectiveness of 86% following two doses and 75% after a single dose, with breakthrough infection rates below 1%. Postexposure prophylaxis with two doses demonstrates effectiveness ranging from 66% to 86% [68]. However, some studies indicate that MVA-BN may elicit only a modest immune response against MPX, suggesting that its protective efficacy requires further evaluation [69].

## 12. Discussion

The genetic diversity of MPXV is profoundly shaping the trajectory of the global MPX outbreak. Although the first human case was not identified until 1970, it was initially misdiagnosed as smallpox due to symptomatic similarities and limited medical and diagnostic capabilities at the time. Whether human infections occurred prior to 1970 remains unknown and is difficult to corroborate. However, based on molecular analyses of infection mechanisms shared among MPXV and other orthopoxviruses in human cells, it is plausible that MPXV had already acquired the molecular determinants necessary for human infection before 1970. Since 1970, reported human cases have emerged initially across Africa and subsequently worldwide, yet effective measures to achieve global eradication remain elusive. A newly identified subclade, Ib, detected in eastern Democratic Republic of the Congo in 2024, exhibits enhanced human-to-human transmissibility, with children under 15 years of age representing the most affected group. This is largely attributable to their lack of smallpox vaccination and consequent absence of cross protective immunity. In contrast, although clade IIb responsible for the 2022 global pandemic has a CFR of only 0.1%, it demonstrated high transmission efficiency within MSM populations, primarily through sexual contact. This shift in transmission dynamics underscores the adaptive potential of MPXV to changing human social behaviors, suggesting that containment efforts may be more complex than initially anticipated.

Furthermore, the immune evasion strategies of MPXV are closely linked to its expanding host range. The OMCP encoded by Clade I exhibits approximately 30% higher affinity for the NKG2D receptor compared to that of Clade II, which may contribute to the increased pathogenicity observed in children, whose NK cell function is not fully mature. Additionally, viral neutralization of IL-1β through viral IL-1β binding proteins (vIL-1βBPs) not only suppresses inflammatory responses but also impairs CD8^+^ T cell activation. This effect is particularly pronounced in people with HIV coinfection, leading to prolonged disease duration and an elevated risk of secondary infections. Clinical profiles of pediatric cases in the Democratic Republic of the Congo in 2024 revealed mucosal involvement in rash lesions in up to 78% of patients, suggesting that the virus may breach primary immune defenses by targeting mucosal immunity, potentially through pathways such as C-X-C chemokine ligand 10 (CXCL10)-mediated chemotaxis. This mechanism is consistent with evolutionary acquisitions in Clade I, including mutations in the M2 protein that enhance suppression of the interferon-gamma stimulated factor 3 (ISGF3) complex, further facilitating immune evasion.

On the other hand, the protective spectrum of existing vaccines requires urgent optimization. The smallpox vaccine confers only 65% cross-protection against Clade I MPXV (compared to 85% against Clade II), with significant waning of immunity beyond 10 years, which explains the high susceptibility observed among children in the Democratic Republic of the Congo. mRNA vaccine development has focused on the A35R protein, a clade I-specific antigen; however, clinical data indicate a 40% reduction in neutralizing antibody binding capacity against emerging Ib subclade strains, underscoring the need for adjuvants that enhance Th1-type immune responses. In terms of therapeutics, the half-maximal effective concentration (EC_50_) of Tecovirimat against Clade I is 2.3-fold higher than that against Clade II, suggesting a potential risk of reduced drug susceptibility. Nevertheless, early intervention with IFN-β combined with local debridement has reduced the rate of severe cases to 0.5% (compared to 4.6% in broader African cohorts), supporting the potential of immune activating therapies. Moving forward, vaccines or therapeutics designed to target MPXV-encoded proteins that suppress innate immune pathways—particularly those aimed at enhancing cellular immunity may represent a promising direction for future antiviral strategies.

## 13. Conclusions

MPX continues to pose a persistent threat to global public health, as evidenced by its ongoing worldwide transmission despite the availability of cross-protective vaccines derived from VARV and therapeutic interventions such as Cidofovir. A major obstacle in the clinical management of MPX is the broad spectrum of immune evasion mechanisms employed by MPXV, which subvert both innate and adaptive host immunity. Consequently, a pivotal future strategy in combating MPX lies in deciphering this intricate immunomodulatory network and its associated physiological processes. This knowledge will be instrumental in developing next-generation vaccines and targeted therapies capable of effectively countering viral immune evasion and robustly eliciting cellular immune responses.

## Data Availability

The data presented in this study are derived from publicly available information published by the WHO and reported in the relevant literature; no new data were generated in this work.

## Supplementary Materials

The following supporting information can be downloaded at: https://ourworldindata.org/mpox#explore-our-data-on-mpox (accessed on 31 July 2025).

## Abbreviations

The following abbreviations are used in this manuscript:

WHO	World Health Organization
MPXV	Mpox virus
MPX	Mpox
PHEIC	Public Health Emergency of International Concern
VARV	Variola virus
VACV	Vaccinia virus
CFR	Case fatality rate
CPXV	Cowpox virus
ORF	Open reading frames
ITR	Inverted terminal repeats
MSM	Men who have sex with men
SNP	Single-nucleotide polymorphisms
IR	Inverted repeat
COG	Conserved oligomeric Golgi
IEV	Intracellular enveloped virion
AR	Androgen receptor
EV	Enveloped virus
MV	Mature virion
CCP	Complement control proteins
TLR	Toll-like receptors
PRR	Pattern recognition receptors
CLR	C-type lectin receptors
RLR	RIG-I-like receptors
NLR	NOD-like receptors
ALR	AIM2-like receptors
cGAS	Cyclic GMP-AMP synthase
IRF	Interferon regulatory factor
MHC	Major histocompatibility complex
NAAT	Nucleic acid amplification test
RT-PCR	Reverse transcription polymerase chain reaction
NEAR	Nicking endonuclease amplification reaction
TMA	Transcription-mediated amplification
LAMP	Loop-mediated isothermal amplification
CRISPR	Clustered regularly interspaced short palindromic repeat
SDA	Strand displacement amplification
qPCR	Real-time quantitative polymerase chain reaction
RPA	Recombinase polymerase amplification
LOD	Limit of detection
ELISA	Enzyme linked immunosorbent assay
IFA	Immunofluorescence assay
FDA	U.S. Food and Drug Administration
CDV-PP	Cidofovir diphosphate
APC	Antigen-presenting cells
MVA	Modified Vaccinia Ankara
Strict	Strict Molecular Clock
UCLN	Uncorrelated Log-Normal Molecular Clock
UCED	Uncorrelated Exponential Molecular Clock
MOPICE	Mpox inhibitor of complement enzymes
APOBEC3	Apolipoprotein B mRNA editing catalytic polypeptide-like 3
OMCP	Orthopoxvirus major histocompatibility complex class I-like protein
HDA	Helicase-dependent amplification
MCP-1	Monocyte Chemoattractant Protein 1
CXCL10	C-X-C Chemokine Ligand 10
ISGF3	Interferon-Gamma Stimulated Factor 3
